# A network analysis of symptom clusters and core symptoms in colorectal cancer patients undergoing postoperative chemotherapy

**DOI:** 10.3389/fonc.2026.1751009

**Published:** 2026-05-06

**Authors:** Bingbing Xiao, Wendan Jing, Jiayi Wang, Jing Zhao, Tingting Tan, Xiangzhen Liu, Qijun Lv, Hongyan Kou

**Affiliations:** 1 Sichuan Branch of National Clinical Research Center for Digestive Diseases, Affiliated Hospital of North Sichuan Medical College, Nanchong, China; 2 Sichuan Clinical Research Center for Digestive Diseases, Affiliated Hospital of North Sichuan Medical College, Nanchong, China; 3 North Sichuan Medical College, Nanchong, China

**Keywords:** colorectal neoplasms, core symptom, drug therapy, network analysis, symptom cluster

## Abstract

**Objective:**

To identify symptom clusters in patients after colorectal cancer surgery upon completion of six cycles of chemotherapy and to determine core symptoms using network analysis, thereby providing evidence for developing precise symptom management strategies.

**Methods:**

A cross-sectional study was conducted. A convenience sample of 211 patients who had completed six cycles of chemotherapy after colorectal cancer surgery was recruited from two tertiary hospitals between February and October 2025. Participants were assessed using the MD Anderson Symptom Inventory for Gastrointestinal Cancer (MDASI-GI). Symptom clusters were extracted via exploratory factor analysis, and a symptom network was constructed using R software to identify core symptoms.

**Results:**

Three symptom clusters were identified: gastrointestinal-psychological, neurotoxicity, and CRC-specific. Network analysis revealed that “Poor appetite” had the highest node strength (*r*_s_=1.362) and expected influence (*r*_e_=1.362), while “Nausea” had the highest closeness centrality (*r*_c_=0.007) and betweenness centrality (*r*_b_=48). Fatigue had the highest incidence rate (89.6%).

**Conclusion:**

Poor appetite and nausea are key symptoms in the symptom network of colorectal cancer patients after surgery and chemotherapy. In clinical practice, giving priority to managing these two core symptoms, while routinely addressing fatigue as a high-frequency concern, may improve patients’ overall symptom experience more effectively and enable more focused care.

## 1 Introduction

Colorectal cancer (CRC) ranks among the most common malignant tumors of the digestive system. According to 2022 global data (1), CRC accounts for 9.6% of all cancer cases, making it the third most prevalent cancer worldwide. In terms of treatment, CRC patients typically undergo comprehensive therapy centered on surgical resection, supplemented by chemotherapy. Most patients begin adjuvant chemotherapy around 4 weeks after surgery to consolidate therapeutic outcomes and prevent cancer recurrence (2). However, under the combined stress of surgery and chemotherapy, patients often experience multiple concurrent physiological and psychological symptoms, such as pain and fatigue, which significantly impair their quality of life (3, 4).

To gain a deeper understanding of the complex relationships among symptoms, methodological approaches in symptom research have evolved from traditional exploratory factor analysis toward network analysis. Exploratory factor analysis, grounded in classical test theory, operates on the core assumption that the co-occurrence patterns of observed symptoms are jointly driven by one or more underlying, unobservable “latent variables” (5). In contrast, symptom network analysis explicitly rejects the concept of latent variables. Its core premise is to treat each symptom as an independent node capable of directly interacting with others, modeling the direct associations between symptoms as network connections. Symptom clusters are conceptualized as sets of closely interconnected symptoms within the network (6). This approach not only provides a visual representation of the complex symptom relationships but also identifies “central symptoms” – those serving as potential hubs within the network that may exert a global influence – through centrality metrics (7). Therefore, network analysis offers a novel and complementary theoretical lens for understanding the dynamic, direct interaction mechanisms among symptoms.

To date, most domestic studies on symptom clusters in postoperative CRC patients undergoing chemotherapy have focused on the early treatment phases (e.g., after the first or third cycle of chemotherapy). Due to variations in assessment tools, statistical methods, and the dynamic evolution of symptoms themselves, there is considerable heterogeneity in the reported structures of symptom clusters and the central symptoms identified (8–10). Furthermore, there is a notable lack of research examining core symptom characteristics at the critical time point after completion of six cycles of chemotherapy (11).

Therefore, this study aims to investigate the symptom network in patients who have completed six cycles of postoperative chemotherapy, with the goal of identifying symptom clusters and central symptoms during this specific period. The findings are expected to provide critical targets and evidence for implementing precise symptom management and improving patients’ quality of life.

## 2 Subjects and methods

### 2.1 Study participants​

This study employed a cross-sectional survey design. From February to October 2025, patients who had undergone surgery for CRC were recruited via convenience sampling from two tertiary-grade class-A hospitals in Nan-chong City, China (specifically from the oncology department, gastrointestinal surgery department, and day chemotherapy center). The inclusion criteria were as follows: (1) diagnosis of CRC confirmed by colonoscopy (2); (2) having undergone radical resection or surgical excision for CRC; (3) completion of six cycles of postoperative adjuvant chemotherapy; (4) age ≥ 18 years; (5) being conscious and providing informed consent; and (6) ability to complete the questionnaires independently or with the assistance of the investigator. The exclusion criteria were: (1) a history of other malignant tumors or severe psychiatric disorders; and (2) poor physical condition precluding participation in the survey.

The sample size was determined based on the number of parameters in the planned network model. The MD Anderson Symptom Inventory–Gastrointestinal Cancer Module (MDASI-GI) assesses 18 symptoms. Constructing a symptom network model for these 18 items requires the estimation of 171 parameters in total (comprising 18 threshold parameters and 18×(18-1)/2 = 153 pairwise association parameters) (12). To ensure model reliability, the sample size should be at least equal to the number of parameters. Accounting for a potential 10% attrition rate, a minimum sample size of 190 participants was targeted.

This study was approved by the Hospital Ethics Committee (Approval No.: 2025ER73-1), and written informed consent was obtained from all participants.

### 2.2 Methods

#### 2.2.1 Assessment instruments

##### 2.2.1.1 General information questionnaire

A self-administered General Information Questionnaire was developed by the investigators, with reference to similar studies (12, 13). It collected data on the following domains: Demographic characteristics:​​ gender, age, occupation, place of residence, marital status, educational level, primary type of health insurance coverage, average monthly household income per capita, smoking history, and drinking history. Clinical characteristics:​​ primary tumor location, receipt of preoperative chemotherapy, chemotherapy regimen, receipt of radiotherapy, receipt of targeted therapy, presence of a stoma, presence of distant metastasis, and number of comorbid chronic conditions.

##### 2.2.1.2 The MD Anderson symptom inventory for gastrointestinal cancer

The MDASI-GI was originally developed at the MD Anderson Cancer Center, USA, in 2000 (14) and was subsequently translated into Chinese by Wang et al. in 2004 (15). It is designed to assess the severity of symptoms experienced by patients in the past 24 hours. The inventory consists of two primary sections: the first section evaluates the severity of 13 core cancer-related symptoms and 5 gastrointestinal cancer-specific symptoms, yielding a total of 18 symptoms; the second section assesses the interference of these symptoms with six different aspects of the patient’s daily life. All items are rated on a numerical rating scale from 0 to 10, where 0 indicates “no symptom” or “no interference” and 10 represents the “most severe symptom” or “complete interference.” Thus, higher scores indicate greater symptom severity or interference. The translated version has demonstrated good reliability and validity, with reported Cronbach’s alpha coefficients of 0.84 for the symptom severity section and 0.90 for the symptom interference section.

In the present study, only the 18-item symptom severity section of the MDASI-GI was used for the symptom network analysis. The internal consistency for this section in our sample was satisfactory, with a Cronbach’s alpha coefficient of 0.819.

#### 2.2.2 Data collection

After obtaining approval from the department, the research team distributed questionnaires to participants 2–3 days after their sixth cycle of chemotherapy. The investigator conducted one-on-one surveys with eligible patients, using a standardized script to explain the study purpose and completion instructions. Participants filled out the questionnaires independently, and the completed forms were checked and collected on the spot. For patients who had been discharged and could not complete the survey in person, follow-up was conducted by telephone. To ensure consistency of the measurement tool, an electronic version identical in content to the paper questionnaire was used. During the call, the investigator read each question and its options verbatim to ensure accurate comprehension. If the patient expressed uncertainty, the investigator provided a standardized, non-leading explanation. All responses were accurately recorded, and key answers were reiterated to the patient for confirmation at the end of the interview.

A total of 222 questionnaires were distributed. After excluding 11 due to patterned or identical responses, 211 valid questionnaires were collected, yielding a valid response rate of 95.05%.

#### 2.2.3 Statistical analysis

##### 2.2.3.1 Descriptive statistics

Descriptive analyses were performed using SPSS software (version 27.0). Continuous variables that followed a normal distribution are presented as the mean ± standard deviation, while those with a non-normal distribution are summarized as the median (P25, P75). Categorical variables are described using frequencies and percentages.

##### 2.2.3.2 Exploratory factor analysis for symptom cluster identification

This study employed exploratory factor analysis (EFA) with varimax rotation to extract symptom clusters. To ensure the robustness of the analysis and avoid statistical noise or overfitting resulting from the inclusion of low-frequency symptoms, only symptoms with an incidence rate >20% were included in the analysis, based on previous studies (16). Prior to EFA, the Kaiser-Meyer-Olkin (KMO) measure and Bartlett’s test of sphericity were conducted to assess the suitability of the data for factor analysis. A KMO value greater than 0.5 and a significant Bartlett’s test (p < 0.05) were considered indicative of adequate factorability. Factors were retained based on the following criteria: (1) eigenvalues ≥ 1; (2) containing at least two symptoms; and (3) symptoms with factor loadings ≥ 0.4 on the retained factor. If a symptom exhibited a loading ≥ 0.4 on multiple factors, it was assigned to the factor on which it had the highest loading.

##### 2.2.3.3 Network analysis

Network analysis was performed using the qgraph package in R version 4.4.3, based on the Gaussian Graphical Model (GGM) with regularization and sparsity estimation via the EBICglasso algorithm. The Fruchterman–Reingold force-directed algorithm was applied for network layout. Symptoms were defined as network nodes, connections between nodes represent network edges, and the thickness of the edges reflects the strength of association between nodes.

Central symptoms within the network were identified by assessing three commonly used centrality indices: strength, closeness, and betweenness. Node strength is calculated as the sum of the absolute weights of all edges connected to that node; a higher strength indicates a greater direct influence on other nodes. Closeness centrality is the inverse of the sum of the shortest path lengths from a node to all other nodes, reflecting how efficiently a node can interact with the rest of the network. Betweenness centrality quantifies the number of times a node acts as a bridge along the shortest path between two other nodes (17). The Expected Influence (EI) index, which is the sum of the edge weights (including negative weights) connecting a node to all others, was also computed as a comprehensive measure of a node’s overall influence on network stability; a higher EI indicates greater centrality and importance (18).

The stability and accuracy of the estimated network were evaluated using the bootnetpackage (19). Network stability was assessed via the Correlation Stability Coefficient (CS), which should ideally be above 0.50 and not below 0.25 (20). The accuracy of the edge weights was examined by plotting the 95% confidence intervals (CIs) around the estimated weights; narrower CIs indicate more precise estimates.

## 3 Results​

### 3.1 Demographic and clinical characteristics of postoperative CRC patients undergoing chemotherapy​

A total of 211 patients completed the study. The majority of patients (204, 96.7%) were over 41 years old. There was a higher proportion of males (122, 57.8%) and most participants were married (198, 93.8%). In terms of educational attainment, a significant majority (175, 82.9%) had a junior high school education or below. Occupations were predominantly categorized as farmers or workers (101, 47.9%) **Table 1**.

**Table 1:** General characteristics of colorectal cancer patients undergoing postoperative chemotherapy (N = 211)​.

Variables	Categories	*N*(*%*)	Variables	Categories	*N*(*%*)
Sex	male	122(57.8)	smoking history	Yes	46(21.8)
	female	89(42.2)		No	165(78.2)
Age (years)	≤40	7(3.3)	Alcohol consumption	Yes	45(21.3)
	41~60	92(43.6)		No	166(78.7)
	≥61	112(53.1)	Tumor type	Colon	84(39.8)
Educational level	Primary school or below	95(45.0)		Rectal	118(55.9)
	Secondary school	80(37.9)		Colorectal	9(4.3)
	Post-secondary	24(11.4)	Neoadjuvant chemotherapy	Yes	73(34.6)
	University or above	12(5.7)		No	138(65.4)
marital status	Single	2(0.9)	Chemotherapy regimens*	FOLFOX	88(41.7)
	Married	198(93.8)		XELOX	109(51.7)
	Divorced/widowed	11(5.2)		FOLFIRI	8(3.8)
Current residence	Rural	94(44.5)		Others	6(2.8)
	Urban	85(40.3)	Radiotherapy	Yes	17(8.1)
	Urban-rural fringe	32(15.2)		No	194(91.9)
Occupational status	Employed	27(12.8)	Targeted therapy	Yes	48(22.7)
	Farmer/Worker	101(47.9)		No	163(77.3)
	Retired	29(13.7)	Distant metastasis	Yes	29(13.7)
	Unemployed	54(25.6)		No	182(86.3)
​Health insurance type	Out-of-pocket	15(7.1)	Enterostomy	Yes	41(19.4)
	Employee Basic Medical Insurance	47(22.3)		No	170(80.6)
	Resident Basic Medical Insurance	149(70.6)	Comorbidities**	0	143(67.8)
Monthly household income	<2000CNY	61(28.9)		1	53(25.1)
	2000-5000CNY	92(43.6)		≥2	15(7.1)
	>5000CNY	58(27.5)			

*In the chemotherapy regimen, FOLFOX refers to oxaliplatin + fluorouracil + calcium folinate, XELOX refers to oxaliplatin + capecitabine, and FOLFIRI refers to fluorouracil + calcium folinate + irinotecan; **There are concurrent chronic diseases such as hypertension, diabetes, coronary heart disease, chronic obstructive emphysema, asthma, and kidney disease.

### 3.2 Symptom prevalence, severity, and symptom cluster extraction in postoperative CRC patients undergoing chemotherapy​

The prevalence, severity, and results of symptom cluster extraction are presented in **Table 2**. As shown, the three most frequently reported symptoms were fatigue (89.6%), distress (85.3%), and poor appetite (78.2%). Fourteen symptoms with a prevalence exceeding 20% were included in the factor analysis. The Kaiser-Meyer-Olkin (KMO) measure was 0.819, and Bartlett’s test of sphericity was significant (χ² = 972.745, *P* <.001), indicating the data were suitable for factor analysis. Principal component analysis initially extracted four factors. However, based on the clinical rationale that a symptom cluster must comprise at least two interrelated symptoms, Factor 4, which contained only a single symptom, was excluded. Consequently, three distinct symptom clusters were retained.

**Table 2:** ​​ Results of symptom prevalence, severity, and symptom cluster extraction in postoperative colorectal cancer patients undergoing chemotherapy (N = 211)​.

Variables	Prevalence *n*(%)	Severity	Factor loading			
		*M*(*P*_25_, *P*_75_)	Factor 1	Factor 2	Factor 3	Factor 4
Poor appetite	165(78.2)	4(3, 5)	0.843			
Sadness	158(74.9)	4(0, 4)	0.718			
Fatigue	189(89.6)	5(4, 6)	0.705			
Distress	180(85.3)	4(3, 5)	0.704			
Dysgeusia	143(67.8)	4(0, 5)	0.704			
Drowsiness	135(64.0)	3(0, 4)	0.692			
Nausea	113(53.6)	3(0, 4)	0.688			
Sleep disturbance	148(70.1)	4(0, 5)	0.687			
Dry mouth	127(60.2)	3(0, 4)		0.821		
Numbness	137(64.9)	4(0, 4)		0.736		
Difficulty remembering	108(51.2)	3(0, 4)		0.734		
Diarrhea	103(48.8)	0(0, 4)			0.877	
Constipation	64(30.3)	0(0, 4)			-0.591	0.492
Pain	69(32.7)	0(0, 4)				0.883
Eigenvalue	—	—	30.55	13.39	9.84	9.28
Cumulative variance contribution rate (*%*)	—	—	30.55	43.94	53.78	63.06

**Figure 1:**
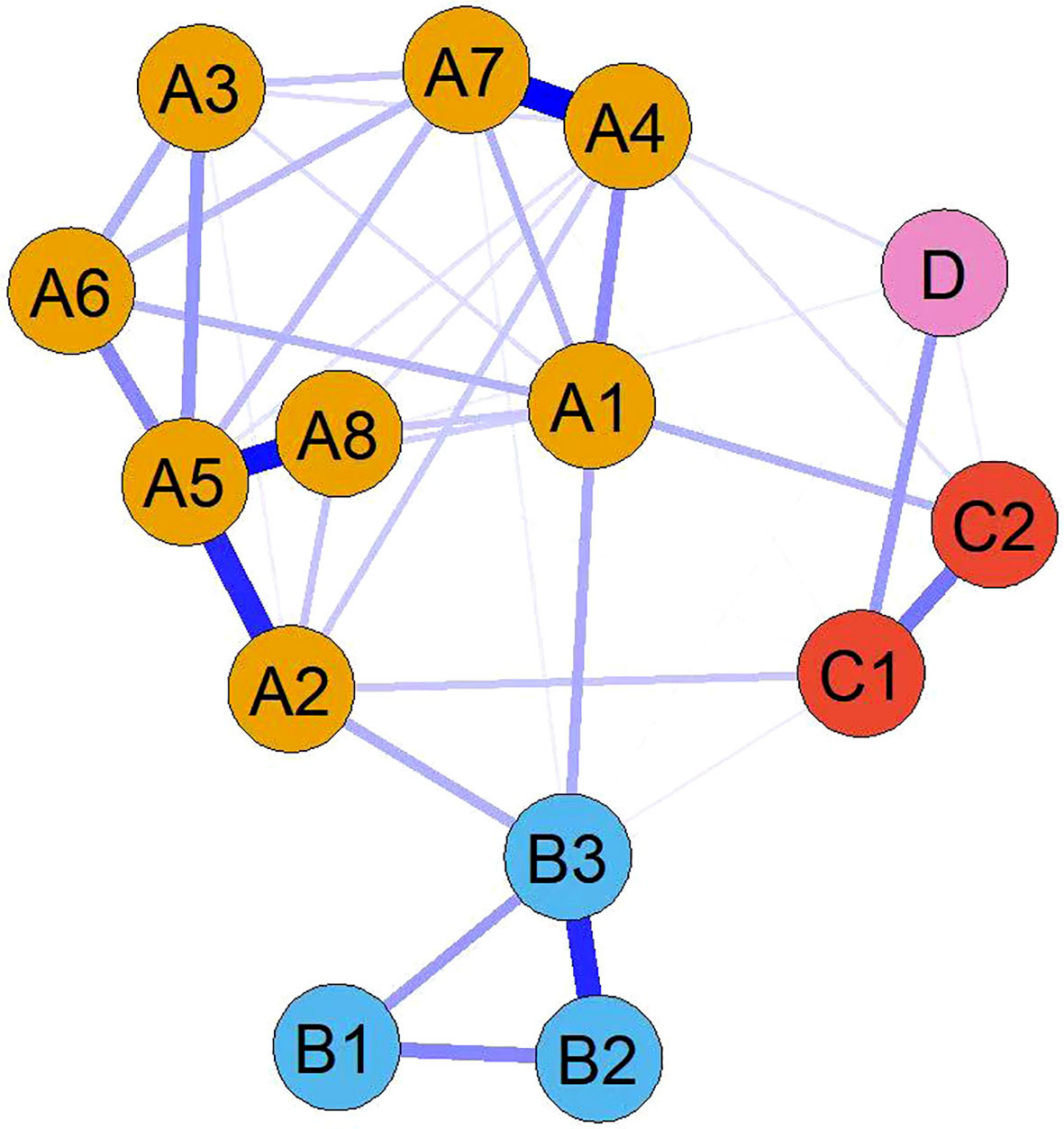
Symptom cluster network of postoperative colorectal cancer patients undergoing chemotherapy. Orange circles (gastrointestinal-psychological cluster): A1 Fatigue, A2 Nausea, A3 Sleep disturbance, A4 Distress, A5 Poor appetite, A6 Drowsiness, A7 Sadness, A8 Taste change. Blue circles (neurotoxicity cluster): B1 Forgetfulness, B2 Dry mouth, B3 Numbness. Red circles (CRC-specific cluster): C1 Constipation, C2 Diarrhea, D Pain.

These clusters were named according to the characteristics of their constituent symptoms: the gastrointestinal-psychological cluster, the neurotoxicity cluster, and the CRC-specific cluster. **Figure 1**, the symptom network diagram, visually represents these three final clusters using different colors. The gastrointestinal-psychological cluster consisted of eight symptoms: nausea, taste change, poor appetite, distress, sadness, fatigue, drowsiness, and sleep disturbance. The neurotoxicity cluster included three symptoms: dry mouth, numbness, and forgetfulness. The CRC-specific cluster was composed of two symptoms: diarrhea and constipation.

### 3.3 Identification of central symptoms in postoperative CRC patients undergoing chemotherapy

**Figure 1**. Symptom cluster network diagram. Each node (circle) represents a symptom, and nodes of the same color belong to the same symptom cluster. The lines (edges) between nodes represent partial correlation coefficients among symptoms; the thickness of an edge indicates the strength of the association (thicker edges represent stronger associations). All edges in the figure are undirected.

**Figure 2:**
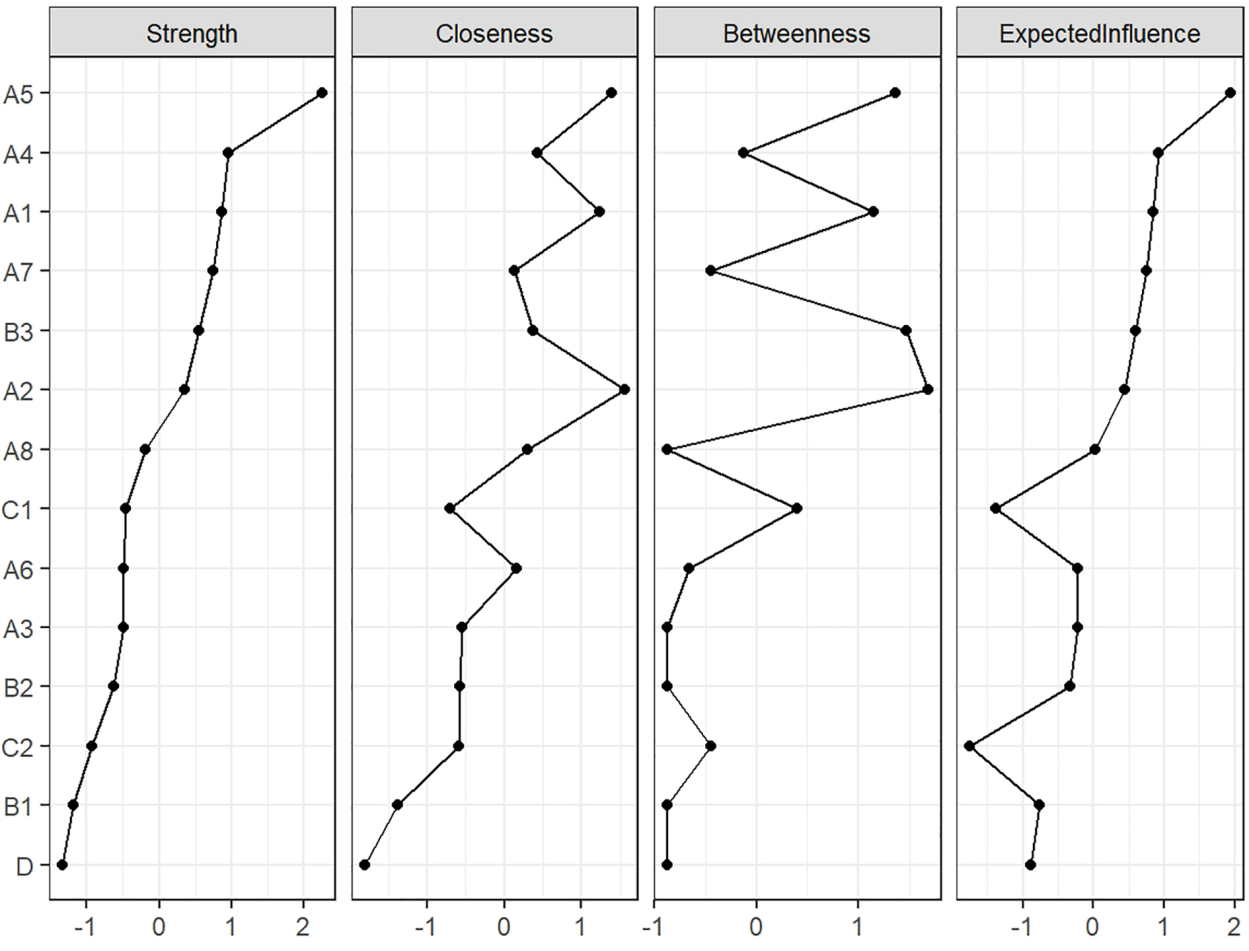
Line plot of centrality indices for the symptom network in postoperative colorectal cancer patients undergoing chemotherapy.

According to the centrality indices shown in **Figure 2**, the top three symptoms ranked by strength centrality and expected influence were poor appetite (rs = 1.362, EI = 1.362), distress (rs = 0.987, EI = 0.987), and fatigue (rs = 0.960, EI = 0.960). The top three symptoms for closeness centrality were nausea (rc = 0.0073), poor appetite (rc = 0.0071), and fatigue (rc = 0.0069). For betweenness centrality, the leading symptoms were nausea (rb = 48), numbness (rb = 44), and poor appetite (rb = 42). Based on these centrality indices, poor appetite was identified as the most central symptom in the network. Furthermore, nausea exhibited the highest closeness centrality, indicating its proximal position within the network and high degree of closeness to other symptoms.

**Figure 3:**
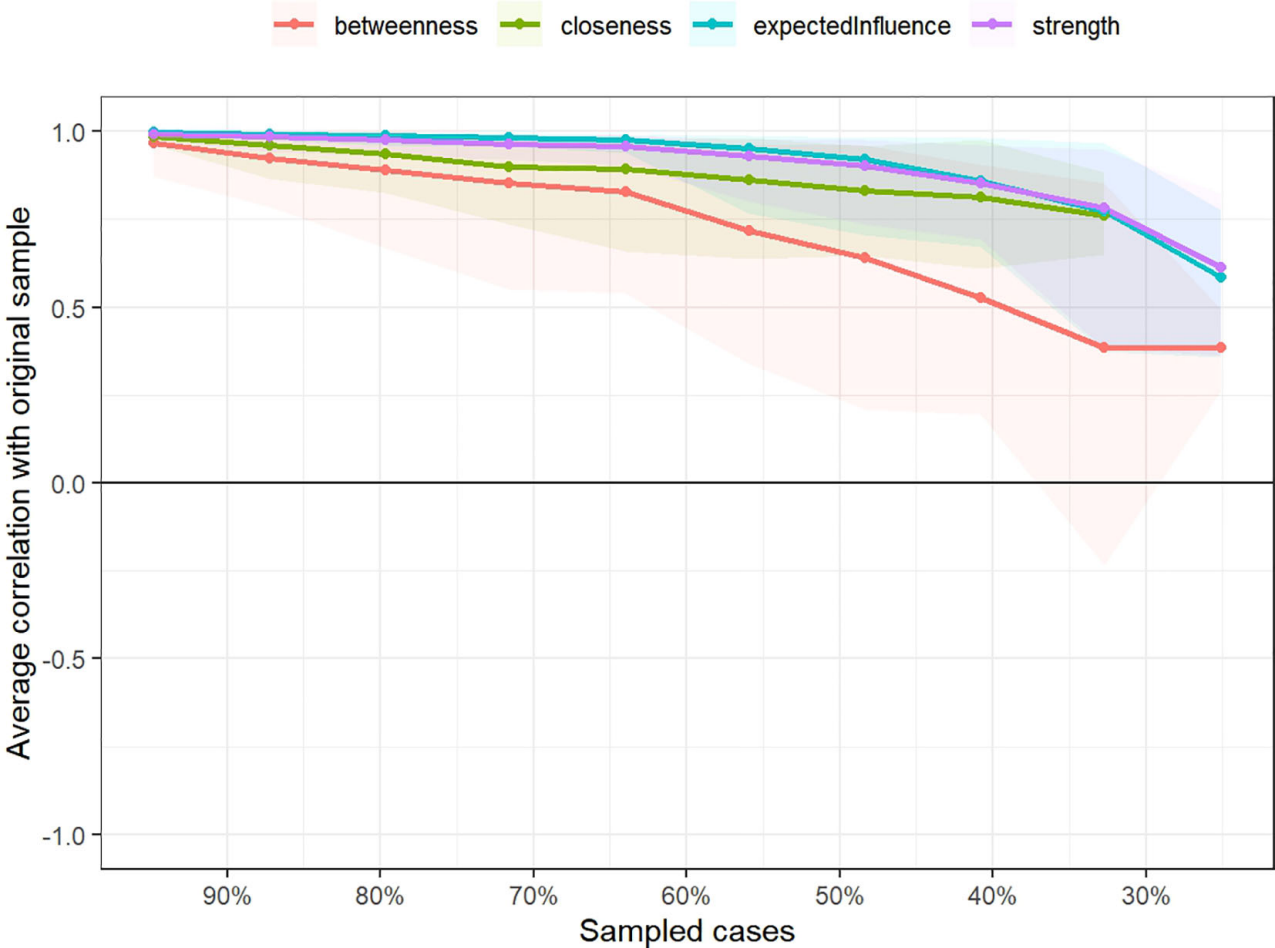
Stability test of the centrality indices for the symptom cluster network analysis in postoperative colorectal cancer patients undergoing chemotherapy.

**Figure 4:**
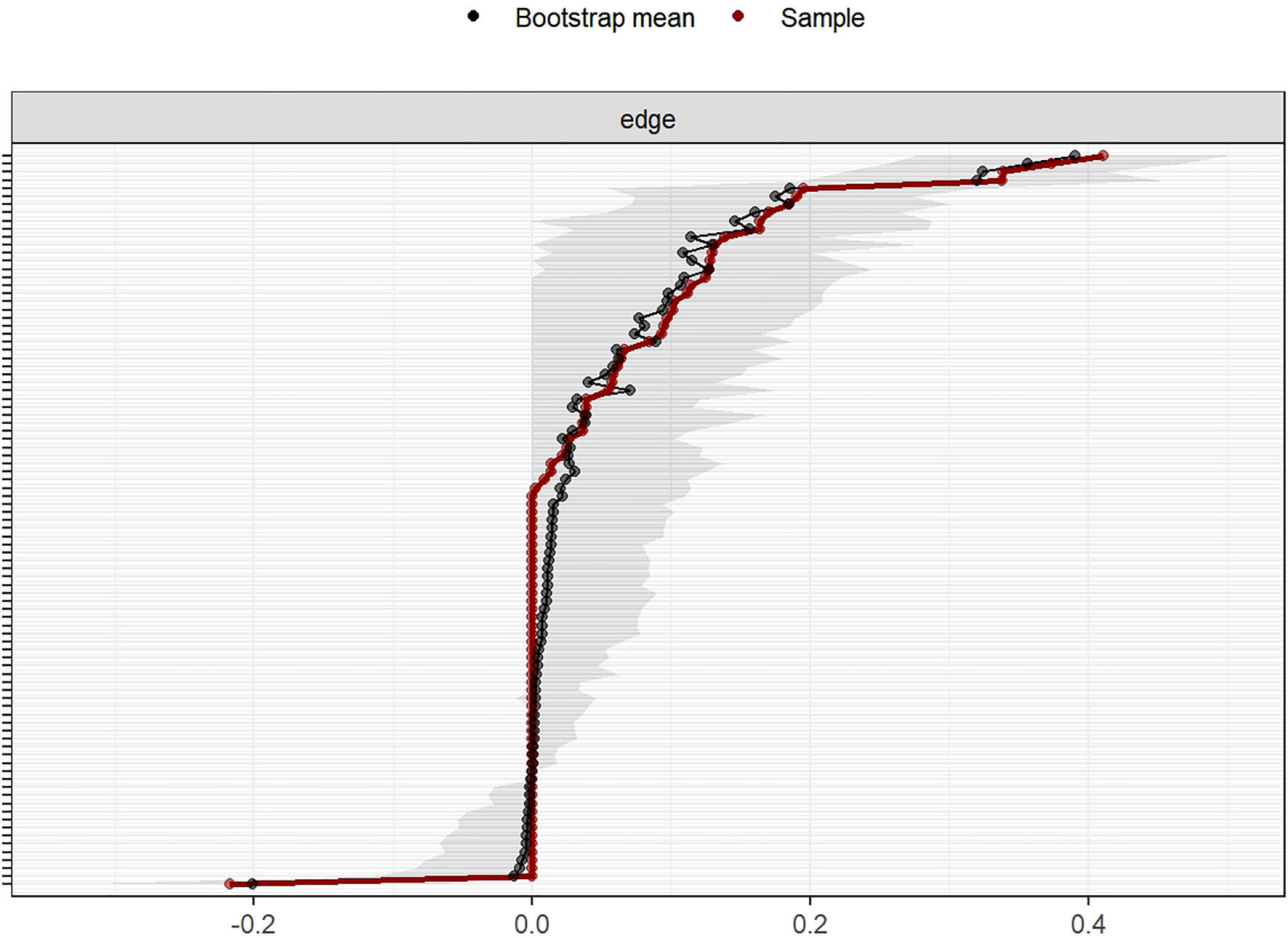
Accuracy test of the centrality indices for the symptom cluster network analysis in postoperative colorectal cancer patients undergoing chemotherapy.

The stability of the network centrality indices, assessed using the bootstrapped correlation stability coefficient (CS), is shown in **Figure 3**. The CS coefficient was 0.517 for strength, 0.517 for expected influence, 0.284 for closeness, and 0.128 for betweenness centrality. The narrow 95% confidence intervals for the edge weights, presented in **Figure 4**, indicate a high accuracy of the network estimation.

## 4 Discussion​

### 4.1 Characteristics of symptom clusters in postoperative CRC patients undergoing chemotherapy

Using principal component analysis, this study identified three distinct symptom clusters: a gastrointestinal-psychological cluster, a neurotoxicity cluster, and a CRC-specific cluster. Symptoms including nausea, taste change, poor appetite, distress, sadness, fatigue, drowsiness, and sleep disturbance are common side effects of chemotherapeutic agents. While chemotherapy aims to prevent cancer dissemination, its associated gastrointestinal reactions, compounded by the inability of some patients with stomas to voluntarily control defecation, often lead to restricted food intake to manage effluent and reduce stoma-related inconvenience. This can result in appetite loss and taste alterations, further impairing nutritional intake and subsequently triggering fatigue and drowsiness. This persistent physiological stress and diminished quality of life can lower psychological resilience, precipitating negative emotions such as distress and sadness. Particularly for patients with postoperative stomas, altered bowel habits significantly impact both physical and mental well-being, often leading to moderate-to-high levels of stigma and sleep disturbances (21). These symptoms are interconnected through underlying physiological-psychological pathways, forming an integrated whole. Factor 1 was thus labeled the gastrointestinal-psychological cluster.

Chemotherapeutic drugs are typically cytotoxic, damaging or inhibiting tumor cells while also harming normal cells to varying degrees, leading to neurotoxic symptoms such as forgetfulness, dry mouth, and numbness. Chemotherapy-induced peripheral neuropathy often manifests as numbness, a direct result of neuronal damage. This neurotoxic effect can extend to the autonomic nervous system, causing dry mouth. Furthermore, the psychological distress associated with chronic neuropathic symptoms, coupled with potential direct central nervous system effects of the drugs, may collectively contribute to cognitive complaints like forgetfulness. Consequently, Factor 2 was named the neurotoxicity cluster.

CRC surgery can damage the intestinal mucosa, reducing its absorptive capacity and predisposing patients to diarrhea. Chemotherapeutic agents can also injure intestinal mucosal cells, leading to functional impairment or dysregulation, resulting in diarrhea or constipation. Therefore, Factor 3 was designated the CRC-specific symptom cluster.

Three symptom clusters were identified in this study, with their specific composition and number showing some divergence from previous research. For instance, Li et al. (22) reported five symptom clusters in postoperative CRC patients receiving chemotherapy, which may be associated with factors such as uneven urban-rural distribution of the sample (75% urban residents), inclusion of patients across multiple chemotherapy cycles (predominantly 1–4 cycles), and a relatively small sample size (n=110). In contrast, the present study focused specifically on patients who had completed a fixed six-cycle chemotherapy regimen after surgery, which may have led to a more concentrated presentation of symptom clusters. Furthermore, differences in assessment tools and statistical approaches could also influence the results. For example, Hu et al. (23) used the Chinese version of the Memorial Symptom Assessment Scale (MSAS-Ch) and identified five symptom clusters by including symptoms with factor loadings ≥0.5. Variations in item content, scale structure, scoring methods, and the cutoff for factor loadings between their study and ours may have contributed to differences in cluster extraction. In summary, the observed variations in symptom clusters are likely attributable to multiple factors, including sample characteristics, assessment instruments, and statistical methods (24, 25). This highlights the importance of carefully considering these methodological differences when comparing or identifying symptom clusters in future research. The present analysis, based on a homogeneous patient population, may offer more targeted insights for symptom management in this specific subgroup.

### 4.2 Analysis of central symptoms in postoperative CRC patients undergoing chemotherapy

Advancements in data analytics provide a novel perspective for understanding the complex interactions of symptoms within a patient’s experience. The identification of central symptoms within a symptom network has become a key focus in network analysis research. Pinpointing these central symptoms facilitates the development of precise interventions targeting the most influential aspects of the symptom network. In this study, network analysis identified poor appetite and nausea as the central symptoms within the symptom clusters of postoperative CRC patients undergoing chemotherapy.

#### 4.2.1 Poor appetite as the symptom with the highest strength centrality

The results of this study show that poor appetite (rs = 1.362) has the highest nodal strength, indicating that it may serve as a central symptom​ in the symptom network of CRC patients undergoing postoperative chemotherapy. Its underlying mechanisms involve the synergistic effects of surgery and chemotherapeutic agents on multiple systems.

Physiologically, chemotherapy drugs can directly damage taste bud cells on the tongue, downregulate the function of taste receptors, trigger abnormal sensations such as metallic taste or heightened bitter sensitivity, and thereby suppress the desire to eat (26). Furthermore, CRC surgery, particularly procedures involving bowel resection and anastomosis, can alter gastrointestinal motility, digestive and absorptive functions, and the secretory rhythm of gut hormones, thereby disrupting the normal regulation of appetite. On the basis of the postoperative state, the cumulative toxicity from the sixth cycle of chemotherapy further exacerbates intestinal mucosal damage, which may induce diarrhea or mucositis, thereby contributing to a series of interrelated symptom experiences (27).

Psychologically, the altered body image, dietary anxieties, and fear of incontinence associated with the postoperative state (e.g., presence of a stoma) constitute significant psychological stressors. This psychological burden, intertwined with the physical discomfort of chemotherapy, depletes the patient’s coping resources and further suppresses the desire to eat (28). Thus, poor appetite can be regarded as a convergence point of physical discomfort and psychological distress in this specific population. Early and integrated interventions targeting this symptom are of critical significance for alleviating the overall symptom burden associated with this central node, improving nutritional status, and potentially enhancing treatment outcomes.

Specifically, in clinical practice, it may be considered to guide patients to adopt dietary strategies such as eating smaller, more frequent meals, prioritizing foods that are nutrient−dense, soft in texture, and mild in flavor. Alternatively, rinsing the mouth before meals or appropriately using natural flavor enhancers such as lemon, mint, or hawthorn may help improve appetite. Meanwhile, it is worthwhile to incorporate dietary anxiety and body image concerns into routine assessment and to investigate the potential value of interventions such as mindful−eating training or cognitive−behavioral therapy in alleviating related psychological distress. Furthermore, within culturally appropriate contexts, the integration of Traditional Chinese Medicine therapies (e.g., herbal formulations or external techniques aimed at regulating spleen−stomach function) as part of a comprehensive management approach could also be explored (29). Through such early, systematic, and multidimensional management, it is anticipated that patients’ nutritional status may be improved, their tolerance to treatment enhanced, and their quality of life elevated.

#### 4.2.2 Nausea as the symptom with the highest closeness centrality

Network analysis in this study revealed that nausea (rc=0.0073) possessed the highest closeness centrality within the symptom network of postoperative CRC patients undergoing chemotherapy. This finding suggests that nausea may not merely be an isolated symptom but rather a central node statistically linked most closely to multiple other symptoms. Its high closeness centrality reflects its position of central association within the symptom network. Based on this pattern of connectivity, interventions targeting nausea might positively influence the overall symptom burden closely connected to it. The mechanism by which nausea becomes such a densely connected central node in this population is closely related to the characteristics of CRC treatment. Firstly, from a pathophysiological perspective, nausea is a direct and early response triggered by chemotherapeutic agents stimulating the vomiting center in the medulla oblongata and inducing neurotransmitter release from enterochromaffin cells in the gut (30). This intense physiological experience often co-occurs with a range of other symptoms. For example, persistent nausea is highly concurrent with symptoms such as poor appetite and taste alterations, potentially contributing to the formation of an interrelated “nausea-anorexia” symptom cluster (31). Simultaneously, severe nausea is also closely associated with vomiting and may influence intestinal function through the brain–gut axis, thereby linking it to symptoms such as diarrhea or constipation (32).

Secondly, for postoperative CRC patients, particularly those with ostomies, the fear of vomiting or loss of bowel control can significantly amplify the anxiety associated with nausea. The distress, sadness, and food aversion that accompany nausea may further deplete the patient’s energy, thereby linking it to symptoms such as exacerbated fatigue and sleep disturbances. This forms a closely intertwined network of “physical discomfort–psychological distress–somatic symptoms,” within which nausea occupies a centrally connected position.

Therefore, the highest closeness centrality of nausea observed in this study strongly indicates its central role in the specific phase of postoperative chemotherapy for CRC. These findings provide an important reference for exploring targeted care strategies: consideration should be given to prioritizing the prevention and management of nausea in symptom interventions. During chemotherapy, evidence-based preventive antiemetic regimens aligned with clinical guidelines (33) can be actively adopted, including standardized use of relevant medications to alleviate nausea and vomiting, combined with non-pharmacological interventions such as progressive muscle relaxation training and individualized music therapy to help reduce these symptoms (34). When such evidence-based routine interventions are integrated with the central symptoms identified through network analysis, they may offer a novel perspective for alleviating the tight interconnections among symptoms and improving patients’ overall experience and quality of life.

#### 4.2.3 Fatigue as the most prevalent symptom

This study found that fatigue (89.6%) was the most frequently reported and severe symptom among postoperative CRC patients undergoing chemotherapy. However, it was not identified as a central symptom in the network analysis, a finding consistent with the results reported by Zheng Cunfeng et al. (10). This suggests that highly prevalent symptoms do not necessarily function as central hubs within the symptom network. For these patients, fatigue is more likely a pervasive background symptom driven by multiple factors. These include the cancer itself and side effects of chemotherapy agents such as oxaliplatin and 5-fluorouracil, which can induce mitochondrial dysfunction and the release of inflammatory cytokines, widely recognized as primary contributors to fatigue (35, 36). Furthermore, fatigue can be exacerbated by the complex interplay with other symptoms such as pain, sleep disturbance, emotional distress, and nutritional deficiencies (11, 37, 38).

Although fatigue was not a central node in our network analysis, its high prevalence and devastating impact on quality of life unequivocally mark it as a critical target for clinical intervention. Non-pharmacological management strategies, particularly exercise and psychological interventions, are commonly recommended for cancer-related fatigue (39). Research has demonstrated that aerobic and resistance training can effectively alleviate fatigue symptoms in these patients (40). Therefore, healthcare providers should proactively and routinely assess fatigue during chemotherapy and integrate its management into the overall supportive care plan. For patients experiencing fatigue, guidance on structured exercise—such as a combination of moderate-intensity aerobic and resistance training three times per week—coupled with psychological interventions like mindfulness-based therapy, can be effective non-pharmacological approaches to reduce fatigue levels (40, 41).

## 5 Conclusion​

This study identified three key symptom clusters—the gastrointestinal-psychological cluster, the neurotoxicity cluster, and the CRC-specific cluster—using a combination of exploratory factor analysis and network analysis. Poor appetite (exhibiting the highest node strength) and nausea (demonstrating the highest closeness centrality) were identified as central symptoms within the symptom network, while fatigue was the most prevalent symptom. Based on these findings, healthcare professionals should prioritize poor appetite and nausea as core targets for intervention. Simultaneously, fatigue should be managed as a routine focus. Developing precise intervention strategies centered on these symptoms is essential for improving intervention efficiency and patient quality of life.

This study also has several limitations. First, as a cross-sectional survey, it cannot reveal dynamic changes in patients’ symptom networks over time or establish causal relationships between symptoms. Future research could explore the longitudinal trajectories of symptom clusters and central symptoms in CRC patients during different phases of postoperative chemotherapy. Second, although the MDASI-GI scale used in this study covers common gastrointestinal and systemic symptoms, its relatively fixed item set may not fully capture other symptoms that are clinically important in this population or that may serve bridging or peripheral roles in the symptom network. This somewhat limits the identification of different symptom roles (e.g., bridge or peripheral symptoms) in the network, thereby potentially affecting the refinement of symptom management strategies. Future studies could conduct more comprehensive network analyses by optimizing assessment tools or supplementing the existing scale with relevant symptom items. Finally, the study focused on the overall symptom network structure of CRC patients after postoperative chemotherapy, which may differ from more detailed network structures at the individual level. Future research could perform subgroup network comparisons to support the implementation of precise symptom management.

## Data Availability

The raw data supporting the conclusions of this article will be made available by the authors, without undue reservation.
